# *CEACAM6* Gene Variants in Inflammatory Bowel Disease

**DOI:** 10.1371/journal.pone.0019319

**Published:** 2011-04-29

**Authors:** Jürgen Glas, Julia Seiderer, Christoph Fries, Cornelia Tillack, Simone Pfennig, Maria Weidinger, Florian Beigel, Torsten Olszak, Ulrich Lass, Burkhard Göke, Thomas Ochsenkühn, Christiane Wolf, Peter Lohse, Bertram Müller-Myhsok, Julia Diegelmann, Darina Czamara, Stephan Brand

**Affiliations:** 1 Department of Medicine II - Grosshadern, Ludwig-Maximilians-University, Munich, Germany; 2 Department of Preventive Dentistry and Periodontology, Ludwig-Maximilians-University, Munich, Germany; 3 Department of Human Genetics, Rheinisch-Westfälische Technische Hochschule, Aachen, Germany; 4 Division of Gastroenterology, Hepatology and Endoscopy, Brigham and Women's Hospital, Harvard Medical School, Boston, Massachusetts, United States of America; 5 TIB MOLBIOL Syntheselabor GmbH, Berlin, Germany; 6 Max-Planck-Institute of Psychiatry, Munich, Germany; 7 Department of Clinical Chemistry, Ludwig-Maximilians-University, Munich, Germany; McGill University, Canada

## Abstract

**Background:**

The carcinoembryonic antigen-related cell adhesion molecule 6 (CEACAM6) acts as a receptor for adherent-invasive *E. coli* (AIEC) and its ileal expression is increased in patients with Crohn's disease (CD). Given its contribution to the pathogenesis of CD, we aimed to investigate the role of genetic variants in the *CEACAM6* region in patients with inflammatory bowel diseases (IBD).

**Methodology:**

In this study, a total of 2,683 genomic DNA samples (including DNA from 858 CD patients, 475 patients with ulcerative colitis (UC), and 1,350 healthy, unrelated controls) was analyzed for eight *CEACAM6* SNPs (rs10415946, rs1805223 = p.Pro42Pro, rs4803507, rs4803508, rs11548735 = p.Gly239Val, rs7246116 = pHis260His, rs2701, rs10416839). In addition, a detailed haplotype analysis and genotype-phenotype analysis were performed. Overall, our genotype analysis did not reveal any significant association of the investigated *CEACAM6* SNPs and haplotypes with CD or UC susceptibility, although certain *CEACAM6* SNPs modulated CEACAM6 expression in intestinal epithelial cell lines. Despite its function as receptor of AIEC in ileal CD, we found no association of the *CEACAM6* SNPs with ileal or ileocolonic CD. Moreover, there was no evidence of epistasis between the analyzed *CEACAM6* variants and the main CD-associated *NOD2*, *IL23R* and *ATG16L1* variants.

**Conclusions:**

This study represents the first detailed analysis of *CEACAM6* variants in IBD patients. Despite its important role in bacterial attachment in ileal CD, we could not demonstrate a role for *CEACAM6* variants in IBD susceptibility or regarding an ileal CD phenotype. Further functional studies are required to analyze if these gene variants modulate ileal bacterial attachment.

## Introduction

Crohn's disease (CD) and ulcerative colitis (UC) are chronic inflammatory bowel diseases (IBD), characterized by an aberrant mucosal immune response to bacteria-derived antigens in the gut of genetically susceptible hosts [1], [2]. Although the exact pathogenesis of IBD still remains unsolved, current evidence indicates that defective T-cell apoptosis [3] and autophagy [4], [5], [6], [7] as well as an impairment of intestinal epithelial barrier function [8], [9] play important roles. This hypothesis is strengthened by data from genetic association studies identifying CD susceptibility genes involved in innate immunity and bacterial recognition (*NOD2/CARD15*) [10], [11], and from genome-wide association studies (GWAS), which identified susceptibility genes involved in autophagy (*ATG16L1*, *IRGM*) [4], [5] and the proinflammatory IL-23/Th17 pathway [12].

While a specific causative pathogen in IBD has not been found so far [13], [14], investigations of the regulatory mechanisms operating at the mucosal level suggest that regulatory cells reactive to the commensal intestinal microflora might play a role in cross-reactive protection toward different bacterial antigens [15]. Moreover, there is raising evidence for a major role of certain bacteria such as adherent-invasive *E. coli* (AIEC) in ileal CD [16], [17], [18]. Interestingly, the carcinoembryonic antigen-related cell adhesion molecule 6 (CEACAM6) has recently shown to act as a receptor for AIEC, supporting ileal bacterial colonization as a major pathomechanism in CD [19]. The carcinoembryonic antigen (CEA) family consists of two subfamilies, the CEACAM subgroup and the pregnancy specific glycoprotein (PSG) subgroup [20], [21]. CEACAM family members were found to be expressed in epithelial, endothelial, and hematopoietic cells, including T-lymphocytes, natural killer (NK) cells, dendritic cells (DC) and neutrophils. They may also be useful as biomarkers in cancer since they are often over-expressed in ovarian, endometrial, breast, lung, and colon carcinomas [21], [22], [23]. Depending on the tissue involved, CEACAMs are transmitting signals that result in a variety of effects including regulation of the cell cycle, tumor suppression, angiogenesis, lymphocyte activation and adhesion [22], [23], [24], [25], [26], [27], [28], [29]. CEACAM1, CEACAM5, and CEACAM6 represent three of the CEACAM subfamily members expressed in intestinal epithelial cells. There is increased expression of CEACAM5 and CEACAM6 at the apical surface of the ileal epithelium in CD patients [19]. Moreover, ileal lesions in CD patients were found to be colonized by pathogenic AIEC [19], strengthening the hypothesis that an abnormal intestinal expression of CEACAM6 in CD patients is associated with an increased colonization of AIEC via type 1 pili expression inducing gut inflammation [18]. AIEC adhere to and invade intestinal epithelial cells [30] resulting in AIEC accumulation in macrophages leading to high amounts of TNF-α [31], thereby perpetuating intestinal inflammation.

**Figure 1:**
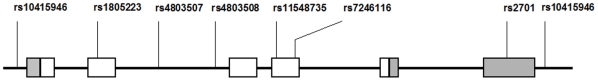
Exon-intron structure of the *CEACAM6* gene and relative positions of single nucleotide polymorphisms (SNPs) investigated in the presented study. This figure represents the genomic structure of the *CEACAM6* gene consisting of 6 exons and indicates the positions of the *CEACAM6* SNPs studied. The SNPs rs4803507 and rs4803508 are localized in intron 2, rs2701 is localized in exon 6 encoding the 3′-untranslated region, while the SNPs rs10415946 and rs10416839 are within the 5′- and the 3′-flanking region, respectively. The coding variant rs1805223 = p.Pro42Pro is located in exon 2, while rs11548735 = p.Gly239Val and rs7246116 = pHis260His are located in exon 4. The grey part of exons 1 represents the 5′ untranslated region, the grey part of exons 5 and exon 6 represent the 3′ untranslated region.

Given the potential implication of dysfunctional CEACAM6 expression in the pathogenesis of IBD, we aimed to analyze the role of *CEACAM6* SNPs in IBD susceptibility. A total of eight single nucleotide polymorphisms (SNPs) were analyzed in a large German cohort of CD and UC patients. Five SNPs in the *CEACAM6* region (rs10415946, rs4803507, rs4803508, rs2701, rs10416839) were selected from the data of the international HapMap project covering the *CEACAM6* gene plus 10 kB flanking the centromeric and telomeric end of the gene, respectively. Additional selection criteria for the SNPs were a minor allele frequency of at least 5% and a r^2^ of 1. The SNPs rs4803507 and rs4803508 are localized in intron 2, rs2701 is localized within exon 6 encoding the 3′-untranslated region, while the SNPs rs10415946 and rs10416839 are within the 5′- and the 3′-flanking region, respectively. Additionally, the coding variants rs1805223 = p.Pro42Pro (exon 2), rs11548735 = p.Gly239Val and rs7246116 = pHis260His (exon 4) were investigated for which allele frequencies are published und which display a minor allele frequency of at least 5% in the Caucasian population. The structure of the *CEACAM6* gene and the localization of the SNPs investigated in the presented study are shown in figure 1. Considering the abnormal expression of CEACAM6 in the ileal epithelium of CD patients and its role as receptor for ileal AIEC [19], we also analyzed for a potential association with an ileal CD phenotype and investigated potential gene-gene interactions with the *NOD2* gene, which has been shown to be a strong predictor of ileal CD, as well as with other CD susceptibility genes such as *IL23R* and *ATG16L1*.

## Materials and Methods

### Ethics statement

The study was approved by the local Ethics committee of the Ludwig-Maximilians-University of Munich (Department of Medicine) and adhered to the ethical principles for medical research involving human subjects of the Helsinki Declaration. Prior to the study, we obtained written, informed consent from all patients included.

### Study population and characterization of disease phenotype

The study population comprised 858 patients with CD, 475 patients with UC, and 1350 healthy, unrelated controls of Caucasian origin. The study was approved by the local Ethics committee of the Ludwig-Maximilians-University of Munich (Department of Medicine) and adhered to the ethical principles for medical research involving human subjects of the Helsinki Declaration. Prior to the study, we obtained written, informed consent from all patients included. The phenotypic assessment was performed blind to the results of the genotypic data and included demographic data and clinical parameters (behaviour and anatomic location of IBD, disease-related complications, previous surgery or immunosuppressive therapy) which were recorded by investigation of patient charts and a detailed questionnaire. The diagnosis of CD or UC was based on established international guidelines including endoscopic, radiological, and histopathological criteria [32], [33]. Patients with CD were assessed according to the Montreal classification [33] based on age at diagnosis (A), location (L), and behaviour (B) of disease. In patients with UC, anatomic location was also assessed in accordance to the Montreal classification [33], using the criteria ulcerative proctitis (E1), left-sided UC (distal UC; E2), and extensive UC (pancolitis; E3). Patients with indeterminate colitis were excluded from the study. The demographic and phenotypic data of the IBD study population are summarized in Table 1.

**Table 1:** Demographic and phenotypic disease characteristics of the study population.

	Crohn's disease*n = 858*	Ulcerative colitis*n = 475*	Controls*n = 1350*
**Gender**			
Male (%)	45.3	47.9	62.6
Female (%)	54.7	52.5	37.4
**Age** (yrs)			
Mean ± SD	40.2±13.2	42.4±14.4	45.8±10.7
Range	11–81	7–86	18–71
**Body mass index**			
Mean ± SD	23.1±4.2	23.9±4.1	
Range	13–40	15–41	
**Age at diagnosis** (yrs)			
Mean ± SD	27.7±11.8	32.0±13.3	
Range	1–78	9–81	
**Disease duration** (yrs)			
Mean ± SD	11.9±8.6	10.5±7.7	
Range	0–44	1–40	
**Positive family history of IBD** (%)	16.0	16.1	
**Disease localization (Crohn's disease)**			
*n = 764* *			
**L1** (ileal)	113		
**L2** (colonic)	97		
**L3** (ileocolonic)	554		
**+ L4** (upper GI)**	88		
**Disease behaviour (Crohn's disease)**			
*n = 754* *			
**B1** (non-stricturing, non-penetrating)	187		
**B2** (stricturing)	208		
**B3** (penetrating)	359		
**Disease extent (Ulcerative colitis)**			
*n = 260* *			
**E1** (proctitis)		24	
**E2** (left-sided UC)		96	
**E3** (pancolitis)		140	

Disease localization and disease behaviour for Crohn's disease and the disease extent in ulcerative colitis are given according to the Montreal classification of inflammatory bowel diseases.

*Given is the number of patients for which the corresponding disease phenotype information was available.

**Additional upper GI involvement.

### DNA extraction and genotyping of the *CEACAM6* variants

Blood samples were taken from all participants of the study and genomic DNA was isolated from peripheral blood leukocytes using the DNA blood mini kit from Qiagen (Hilden, Germany) according to the manufacturer's guidelines. Eight *CEACAM6* SNPs (rs10415946, rs1805223 = p.Pro42Pro, rs4803507, rs4803508, rs11548735 = p.Gly239Val, rs7246116 = pHis260His, rs2701, rs10416839) were genotyped by PCR and melting curve analysis using a pair of fluorescence resonance energy transfer (FRET) probes in a LightCycler® 480 Instrument (Roche Diagnostics, Mannheim, Germany) as described in previous studies [34], [35], [36]. The total volume of the PCR was 5 µl containing 25 ng of genomic DNA, 1× Light Cycler 480 Genotyping Master Mix (Roche Diagnostics), 2.5 pmol of each primer and 0.75 pmol of each FRET probe (TIB MOLBIOL, Berlin, Germany). In the case of rs1805223, rs4803507 and rs4803508, the concentration of the forward primer, and in the case of rs10415946 and rs2701, the concentration of the reverse primer, were reduced to 1.25 pmol. Two SNPs were analyzed in a multiplex reaction, the combinations were: rs10415946+rs4803508, rs1805223+rs4803507, rs11548735+rs7246116 and rs2701+rs10416839. For the combination rs11548735+rs7246116 only one primer pair was used. The PCR comprised an initial denaturation step (95°C for 10 min) and 45 cycles (95°C for 10°C sec, 60 for 10 sec, 72°C for 15 sec). Details on the melting curve analysis and on the PCR used for sequencing were published in previous studies [34], [35], [36]. The PCR products were purified using the QIAquick PCR Purification Kit (Qiagen) and sequenced by a commercial sequencing company (Sequiserve, Vaterstetten, Germany). All sequences of primers and FRET probes and primer annealing temperatures used for genotyping and for sequence analysis are given in Tables 2 and 3.

**Table 2:** Primer sequences (F: forward primer, R: reverse Primer) and FRET probe sequences used for genotyping of *CEACAM6* variants.

Polymorphism	Primer sequences	FRET probe sequences
rs10415946	F: AGCCCTGGATGTGTCCACA: AGTCCCTGGGGTCCTCAA	TGGATTTACCCCCAGCAAG-FLLC670-AGGTCACAGAGATGTTTGGGGTCCTAG
rs1805223 = p.Pro42Pro	F: CCACCCTAATGCATAGGTCCA: CGATTCTGTGGCAGGTTGT	GAATCCACGCCATTCAATG-FLLC670-CGCAGAGGGGAAGGAGGTTCTTC
rs4803507	F: GCATCGTTCCTTCCTTTATGTAA: TTTTTCCATAAGTGGAGATCGTT	GAATTCACAACACACCTAAACC-FLLC640-AGTATGTTATCAAGAAAAATACTACTTCCAGCCC
rs4803508	F: CCTGTCCCCCTCACTGTCTA: TTTTTCCATAAGTGGAGATCGTT	CTGCTGAAAGATCCAATCCC-FLLC610-GCCAGGCTGCACAGTATCCTTGGG
rs11548735 = p.Gly239Val	F: TGGTTGAGACTTCAGGGTTGTA: TATGGGCTTGGCACATATAGG	CCCAGATGTCCCCACCAT-FLLC610-TCCCCCTCAAAGGCCAATTACCGTC
rs7246116 = pHis260His	F: TGGTTGAGACTTCAGGGTTGTA: TATGGGCTTGGCACATATAGG	CCTGCCACGCAGCCTCTA-FLLC670-CCCACCTGCACAGTACTCTTGGTTTATCAA
rs2701	F: AAGATGTCAAAACAAGACTCCTCAA: AAGTCCAACTCTGAAAAGGACC	CAAGATAGATCTGACACTCTGTTAAGT-FLLC610-ACCCTCTGAAGCTACTTCTTGTGAAATACT
rs10416839	F: CTTTCAGTTATATGTTGGCTCACTTA: AAAAACACAGCATTATAGATCAACAG	CCAGTGGCAGTTTCCTCTG-FLLC640-TGTAGTCTGAATCAGGTGTACAACTGAGCC

Note: FL: Fluorescein, LC610: LightCycler-Red 610; LC640: LightCycler-Red 640. The polymorphic position within the sensor probe is underlined. A phosphate is linked to the 3′-end of the acceptor probe to prevent elongation by the DNA polymerase in the PCR.

**Table 3:** Primer sequences used for the sequence analysis of the *CEACAM6* variants.

Polymorphism	Primer sequences
rs10415946	TGCAGAAAGAACAATTCAGAATCTTA CTTGGGTCTGTCAGCACC
rs1805223 = p.Pro42Pro	GGGTGAAGAGACCTGCTCAG CGCCTTTGTACCAGCTGTAAC
rs4803507	ACGTTGCTTCTAATTTGGCA GAAAAGTTTGTCAGGAGTTTAGACC
rs4803508	CCTGTCCCCCTCACTGTCT ATGGGTGATGATGGGACTTC
rs11548735 = p.Gly239Val, rs7246116 = pHis260His	TGGTTGAGACTTCAGGGTTGT TATGGGCTTGGCACATATAGG
rs2701	AAGATGTCAAAACAAGACTCCTCA AGAACAGGTGAGTCTAGAAGTCCA
rs10416839	CTTTCAGTTATATGTTGGCTCACTT AAAAACACAGCATTATAGATCAACAG

Genotyping data of the three main CD-associated *NOD2* variants p.Arg702Trp (rs2066844), p.Gly908Arg (rs2066845), and p.Leu1007fsX1008 (rs2066847) were available from previous studies [34], [37]. Similarly, for epistasis analysis genotype data for the main CD-associated *IL23R* variants (rs1004819, rs7517847, rs10489629, rs2201841, rs11465804, rs11209026 (p.Arg381Gln), rs1343151, rs10889677, rs11209032, rs1495965) and *ATG16L1* SNPs (rs13412102, rs12471449, rs6431660, rs1441090, rs2289472, rs2241880 (p.Thr300Ala), rs2241879, rs3792106, rs4663396) were available from previous studies [34]–[36].

### RNA isolation and quantitative PCR

Total RNA was isolated from five intestinal epithelial cell (IEC) lines (DLD-1, HCT116, HT-29, SW480, T84) as indicated with the Qiagen RNeasy Kit and was reverse transcribed using Roche Transcriptor First Strand cDNA Synthesis Kit. Quantitative PCR was performed with SYBR Green PCR Master Mix from Roche on a LightCycler480 instrument. The following primers were used for amplification: *CEACAM6* forward 5′-CACAACCTGCCCCAGAATCGTAT-3′; *CEACAM6* reverse 5′-TTGGGCAGCTCCGGGTATACATG-3′; *β-actin* forward 5′-GCCAACCGCGAGAAGATGA-3′; *β-actin* reverse 5′-CATCACGATGCCAGTGGTA-3′. β-actin expression was used to normalize gene expression in the respective samples.

### Statistical analyses

Each genetic marker was tested for Hardy-Weinberg equilibrium in the three subgroups of the study population. Fisher's exact test was used for comparison between categorical variables, while Student's t test was applied for quantitative variables. Single-marker allelic tests were performed with Pearson's χ^2^ test. All tests were two-tailed, considering p-values<0.05 as significant. Odds ratios were calculated for the minor allele at each SNP. For multiple comparisons, Bonferroni correction was applied where indicated. Interactions between different polymorphisms were tested using logistic regression in R using the number of minor alleles as predictor variable, therefore implementing an Armitage test of trend. Data were evaluated by using the SPSS 13.0 software (SPSS Inc., Chicago, IL, USA) and R-2.4.1. (http://cran.r-project.org). For haplotype analysis, PLINK v 1.06 (http://pngu.mgh.harvard.edu/~purcell/plink/) was used running a sliding window approach with variation of the window size from 2 to 8 included markers and using the option “hap-logistic”. Linkage disequilibrium (LD) was also analyzed using PLINK.

## Results

### *CEACAM6* variants are not associated with IBD susceptibility

The genotyping success rates were at least 99% for all eight SNPs tested and were comparable between the controls and the CD and UC patients groups. In all three subgroups (CD, UC, and controls), the allele frequencies of the *CEACAM6* SNPs rs10415946, rs1805223 = p.Pro42Pro, rs4803507, rs4803508, rs11548735 = p.Gly239Val, rs7246116 = p.His260His, rs2701, rs10416839 were in accordance with the predicted Hardy-Weinberg equilibrium (Table 4). Overall, we observed no significant differences in the frequencies of the investigated *CEACAM6* SNPs in CD and UC patients compared to healthy controls (Table 4) implicating no significant association of *CEACAM6* variants and IBD susceptibility. Only two patients (both with UC) were minor allele carriers of the rare *CEACAM6* SNP rs7246116 = p.His260His, therefore not allowing a comparative analysis of this SNP regarding CD susceptibility.

**Table 4:** Associations of *CEACAM6* gene markers in the case-control association studies.

SNP	Minor allele	Crohn's disease*n = 858*			Ulcerative colitis*n = 475*			Controls*n = 1350*	HapMap-CEU** *n = 120*
		MAF/HWE	p value	OR [95% CI]	MAF/HWE	p value	OR [95% CI]	MAF/HWE	MAF
rs10415946	G	0.383/0.506	0.21	1.08 [0.96–1.23]	0.338/0.113	0.16	0.89 [0.76–1.04]	0.364/0.906	0.440
rs1805223 = p.Pro42Pro	A	0.309/0.679	0.21	1.09 [0.95–1.24]	0.264/0.235	0.12	0.87 [0.74–1.03]	0.291/0.262	0.342
rs4803507	A	0.307/0.803	0.24	1.08 [0.96–1.24]	0.269/0.454	0.26	0.90 [0.76–1.06]	0.290/0.234	0.280
rs4803508	A	0.377/0.266	0.25	0.95 [0.84–1.08]	0.406/0.622	0.35	1.07 [0.92–1.25]	0.389/0.606	0.358
rs11548735 = p.Gly239Val	T	0.399/0.885	0.62	0.97 [0.86–1.10]	0.422/1	0.44	1.06 [0.92–1.24]	0.407/0.612	0.422
rs7246116 = pHis260His	T	0/*	1.00	-	0.001/1	0.26	-	0/*	unknown
rs2701	G	0.401/0.942	0.62	0.97 [0.85–1.09]	0.423/0.903	0.47	1.06 [0.91–1.23]	0.409/0.778	0.408
rs10416839	T	0.359/0.653	0.24	1.08 [0.95–1.23]	0.371/1	0.11	1.14 [0.97–1.32]	0.341/0.545	0.292

Minor allele frequencies (MAF), p-value for deviation from Hardy-Weinberg equilibrium (HWE), allelic test *P*-values, and odds ratios (OR, shown for the minor allele) with 95% confidence intervals (CI) are depicted for both the CD and UC case-control cohorts. Measurements for linkage disequilibrium (LD) are provided in Tables 11, 12 and 13.

*monomorphic SNP.

**The MAFs in the HapMap-CEU population ( = Utah residents with Northern and Western European ancestry) are derived from the NCBI SNP database (available under http://www.ncbi.nlm.nih.gov/snp).

### *CEACAM6* haplotypes are not associated with CD and UC susceptibility

Considering recent evidence showing that certain *CEACAM6* haplotypes modulate susceptibility to bacterial infections [38], we next performed a detailed haplotype analysis in our IBD cohort. However, as shown in Tables 5 and 6, we could not demonstrated significant associations of *CEACAM6* haplotypes with CD and UC susceptibility.

**Table 5:** Haplotypes of *CEACAM6* SNPs in Crohn's disease (CD) case-control sample and omnibus p-values for association with CD susceptibility.

Haplotype combination	omnibus p-value
rs10415946-rs1805223	0.46
rs1805223-rs4803507	0.40
rs4803507-rs4803508	0.84
rs4803508-rs11548735	0.63
rs11548735-rs7246116	0.85
rs7246116-rs2701	0.78
rs2701-rs10416839	0.36
rs10415946-rs1805223-rs4803507	0.40
rs1805223-rs4803507-rs4803508	0.58
rs4803507-rs4803508-rs11548735	0.74
rs4803508-rs11548735-rs7246116	0.63
rs11548735-rs7246116-rs2701	0.74
rs7246116-rs2701-rs10416839	0.36
rs10415946-rs1805223-rs4803507-rs4803508	0.48
rs1805223-rs4803507-rs4803508-rs11548735	0.67
rs4803507-rs4803508-rs11548735-rs7246116	0.75
rs4803508-rs11548735-rs7246116-rs2701	0.39
rs11548735-rs7246116-rs2701-rs10416839	0.38
rs10415946-rs1805223-rs4803507-rs4803508-rs11548735	0.70
rs1805223-rs4803507-rs4803508-rs11548735-rs7246116	0.67
rs4803507-rs4803508-rs11548735-rs7246116-rs2701	0.59
rs4803508-rs11548735-rs7246116-rs2701-rs10416839	0.21
rs10415946-rs1805223-rs4803507-rs4803508-rs11548735-rs7246116	0.70
rs1805223-rs4803507-rs4803508-rs11548735-rs7246116-rs2701	0.66
rs4803507-rs4803508-rs11548735-rs7246116-rs2701-rs10416839	0.39
rs10415946-rs1805223-rs4803507-rs4803508-rs11548735-rs7246116-rs2701	0.77
rs1805223-rs4803507-rs4803508-rs11548735-rs7246116-rs2701-rs10416839	0.41
rs10415946-rs1805223-rs4803507-rs4803508-rs11548735-rs7246116-rs2701-rs10416839	0.39

Given are the omnibus p-values for the *CEACAM6* haplotype combinations regarding CD susceptibility.

**Table 6:** Haplotypes of *CEACAM6* SNPs in ulcerative colitis (UC) case-control sample and omnibus p-values for association with UC susceptibility.

Haplotype combination	omnibus p-value
rs10415946-rs1805223	0.50
rs1805223-rs4803507	0.60
rs4803507-rs4803508	0.73
rs4803508-rs11548735	0.50
rs11548735-rs7246116	0.65
rs7246116-rs2701	0.55
rs2701-rs10416839	0.60
rs10415946-rs1805223-rs4803507	0.50
rs1805223-rs4803507-rs4803508	0.82
rs4803507-rs4803508-rs11548735	0.73
rs4803508-rs11548735-rs7246116	0.37
rs11548735-rs7246116-rs2701	0.69
rs7246116-rs2701-rs10416839	0.60
rs10415946-rs1805223-rs4803507-rs4803508	0.81
rs1805223-rs4803507-rs4803508-rs11548735	0.83
rs4803507-rs4803508-rs11548735-rs7246116	0.58
rs4803508-rs11548735-rs7246116-rs2701	0.50
rs11548735-rs7246116-rs2701-rs10416839	0.54
rs10415946-rs1805223-rs4803507-rs4803508-rs11548735	0.87
rs1805223-rs4803507-rs4803508-rs11548735-rs7246116	0.76
rs4803507-rs4803508-rs11548735-rs7246116-rs2701	0.55
rs4803508-rs11548735-rs7246116-rs2701-rs10416839	0.39
rs10415946-rs1805223-rs4803507-rs4803508-rs11548735-rs7246116	0.87
rs1805223-rs4803507-rs4803508-rs11548735-rs7246116-rs2701	0.75
rs4803507-rs4803508-rs11548735-rs7246116-rs2701-rs10416839	0.48
rs10415946-rs1805223-rs4803507-rs4803508-rs11548735-rs7246116-rs2701	0.87
rs1805223-rs4803507-rs4803508-rs11548735-rs7246116-rs2701-rs10416839	0.49
rs10415946-rs1805223-rs4803507-rs4803508-rs11548735-rs7246116-rs2701-rs10416839	0.82

Given are the omnibus p-values for the *CEACAM6* haplotype combinations regarding UC susceptibility.

### The *CEACAM6* variants are not associated with an ileal disease phenotype in CD patients

Since CEACAM6 has recently shown to act as a receptor for AIEC, thereby promoting bacterial colonization in ileal CD [19], we further investigated whether *CEACAM6* SNPs are associated with ileal disease in CD patients. Based on a phenotype analysis according to the Montreal classification of IBD [33], the detailed phenotypic data available from a subcohort of 667 CD patients was analyzed for disease localization. None of the investigated *CEACAM6* SNPs was associated with ileal or ileocolonic CD (Table 7). However, we have to acknowledge that the sample size has limited power to detect weak disease associations. For example, based on 667 patients with a L1/L3 disease phenotype, a minor allele frequency of 0.40 and an OR of 1.1, the power is 28.88% to detect an effect on a significance level of 5% (Genetic Power Calculator, http://pngu.mgh.harvard.edu/~purcell/gpc/).

**Table 7:** Associations of *CEACAM6* gene markers with the anatomic location of Crohn's disease (CD) according to the Montreal classification [33].

Anatomic location	rs10415946	rs1805223 = p.Pro42Pro	rs4803507	rs4803508	rs11548735 = p.Gly239Val	rs7246116 = pHis260His	rs2701	rs10416839
**L1 (ileal)** *n = 113*	0.320	0.321	0.559	0.876	0.961	*	0.997	0.566
**L2 (colonic)** *n = 97*	0.854	0.988	0.970	0.611	0.751	*	0.684	0.580
**L3 (ileocolonic)** *n = 554*	0.451	0.759	0.803	0.425	0.684	*	0.691	0.114
**Any ileal involvement** **(L1+L3)** *n = 667*	0.373	0.586	0.689	0.620	0.771	*	0.744	0.117

*P*-values are depicted for the CD case-control cohorts.

*There were no carriers of the minor allele of rs7146116 in the CD and control cohort.

### Analysis for gene-gene interaction with CD-associated *NOD2*, *IL23R* and *ATG16L1* variants

Given the raising evidence for a key role of CEACAM6 in the complex interaction of the mucosal immune system and intestinal bacteria, we next analyzed for potential epistasis between *CEACAM6* SNPs (rs10415946, rs1805223 = p.Pro42Pro, rs4803507, rs4803508, rs11548735 = p.Gly239Val, rs7246116 = pHis260His, rs2701, rs10416839) and the three main CD-associated *NOD2/CARD15* variants p.Arg702Trp (rs2066844), p.Gly908Arg (rs2066845), and p.Leu1007fsX1008 (rs2066847) which have previously shown to be strongly associated with CD and ileal disease localization. However, there was no evidence for epistasis between the *CEACAM6* SNPs and the three analyzed *NOD2/CARD15* variants (Table 8).

**Table 8:** Analysis for gene-gene interactions between *CEACAM6* and *NOD2* variants regarding susceptibility to Crohn's disease (CD).

*CEACAM6*SNPs*NOD2* SNPs	rs10415946	rs1805223	rs4803507	rs4803508	rs11548735	rs7246116	rs2701	rs10416839
**rs2066844** p.Arg702Trp	0.56	0.37	0.39	0.31	0.62	*	0.65	0.77
**rs2066845** p.Gly908Arg	0.06	0.28	0.28	0.54	0.35	*	0.43	0.58
**rs2066847** p.Leu1007fsX1008	0.93	0.80	0.97	0.51	0.71	*	0.56	0.82

p-values for epistasis analysis between *CEACAM6* and *NOD2* SNPs in the CD case-control sample.

*There were no carriers of the minor allele of rs7146116 in the CD and control cohort.

Recently, we demonstrated an association of the *IL23R* SNP rs1004819 (TT homozygous carriers) with ileal CD [34]. Therefore, we also analyzed for potential gene-gene interaction between *CEACAM6* SNPs and the major CD-associated *IL23R* variants. However, we did not find evidence for epistasis between *CEACAM6* and *IL23R* regarding CD susceptibility (Table 9).

**Table 9:** Analysis for gene-gene interaction with *CEACAM6*.and *IL23R* variants regarding susceptibility to Crohn's disease (CD).

*CEACAM6*SNPs*IL23R* SNPs	rs10415946	rs1805223	rs4803507	rs4803508	rs11548735	rs7246116	rs2701	rs10416839
**rs1004819**	0.13	0.31	0.29	0.50	0.46	*	0.52	0.95
**rs7517847**	0.67	0.20	0.31	0.17	0.23	*	0.27	0.81
**rs10489629**	0.64	0.09	0.11	0.97	0.79	*	0.71	0.98
**rs2201841**	0.52	0.42	0.40	0.61	0.50	*	0.46	0.63
**rs11465804**	0.20	0.13	0.19	0.59	0.26	*	0.32	0.76
**rs11209026**	0.08	0.05	0.08	0.35	0.28	*	0.35	0.91
**rs1343151**	0.35	**0.04**	0.06	0.83	0.57	*	0.60	0.64
**rs10889677**	0.55	0.48	0.44	0.77	0.66	*	0.62	0.51
**rs11209032**	0.43	0.52	0.53	0.87	0.99	*	0.89	0.19
**rs1495965**	0.47	0.66	0.58	0.73	0.61	*	0.84	0.32

p-values for epistasis between *CEACAM6*.and *IL23R* SNPs in the CD case-control sample. After Bonferroni correction, the association highlighted in bold did not remain significant.

*There were no carriers of the minor allele of rs7146116 in the CD and control cohort.

In addition, novel findings indicate a major role for *ATG16L1* in Paneth cell development in the terminal ileum. Therefore, we also analyzed for potential epistasis between the *CEACAM6* SNPs and the major CD-associated *ATG16L1* SNPs. However, we were also unable to demonstrate evidence for epistasis between these two genes (Table 10).

**Table 10:** Analysis for gene-gene interaction between *CEACAM6* and *ATGT16L1* variants regarding susceptibility to Crohn's disease (CD).

*CEACAM6*SNPs*ATG16L1* SNPs	rs10415946	rs1805223	rs4803507	rs4803508	rs11548735	rs7246116	rs2701	rs10416839
**rs13412102**	0.89	0.89	0.72	0.89	0.55	*	0.55	0.79
**rs12471449**	0.49	0.44	0.40	0.74	0.58	*	0.56	**0.04**
**rs6431660**	0.53	0.66	0.50	0.87	0.98	*	0.93	0.66
**rs1441090**	0.56	0.32	0.26	0.78	0.89	*	0.95	0.27
**rs2289472**	0.67	0.65	0.49	0.67	0.86	*	0.83	0.57
**rs2241880**	0.84	0.74	0.57	0.64	0.81	*	0.83	0.58
**rs2241879**	0.93	0.81	0.68	0.74	0.73	*	0.72	0.56
**rs3792106**	0.67	0.52	0.44	0.50	0.69	*	0.73	0.83
**rs4663396**	0.81	0.93	0.80	0.56	0.85	*	0.90	0.15

p-values for epistasis between *CEACAM6* and *ATGT16L1* SNPs in the CD case-control sample. After Bonferroni correction, the association highlighted in bold did not remain significant.

*There were no carriers of the minor allele of rs7146116 in the CD and control cohort.

p-values for epistasis between *CEACAM6* and *ATGT16L1* SNPs in the CD case-control sample. After Bonferroni correction, the association highlighted in bold did not remain significant.

^*^There were no carriers of the minor allele of rs7146116 in the CD and control cohort.

### *CEACAM6* genotypes modulate *CEACAM6* expression in intestinal epithelial cell lines

**Figure 2:**
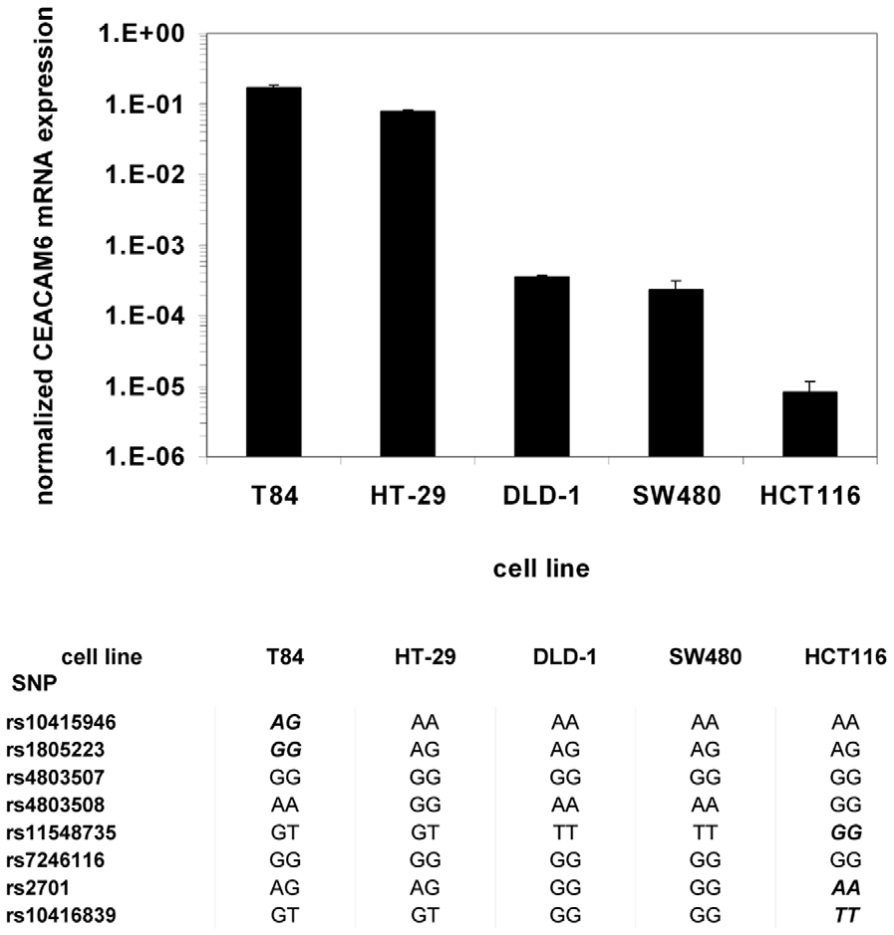
Analysis of CEACAM6 gene expression and *CEACAM6* gene variants in intestinal epithelial cell (IEC) lines. (A) Total RNA isolated from IEC lines as indicated was reverse transcribed and was analyzed for CEACAM6 gene expression by quantitative PCR. T84 cells express CEACAM6 at the highest level followed by HT-29 cells, and intermediate CEACAM6 expression was found in SW480 and DLD-1 cells. CEACAM6 expression was close to the detection limit after 40 PCR cycles in HCT116 cells (note the logarithmic scale on the y-axis). CEACAM6 expression was normalized to β-actin expression in the respective cDNA samples. (B) Genomic DNA was isolated from IEC lines and 8 SNPs in *CEACAM6* were analyzed as indicated by DNA sequencing. The respective alleles for these SNPs in each cell line are depicted in the table. This analysis revealed that T84, the cell line with the highest CEACAM6 expression, and HCT116 cells, the cell line with the lowest CEACAM6 expression, are the only IEC lines with unique genotypes for certain *CEACAM6* SNPs (depicted in bold italic). DLD-1 and SW480 cells have identical genotypes for all SNPs analyzed and nearly identical CEACAM6 expression levels.

To analyze a potential influence of *CEACAM6* gene variants on CEACAM6 gene expression, we determined CEACAM6 mRNA levels in five intestinal epithelial cell (IEC) lines DLD-1, HCT116, HT-29, SW480 and T84 by quantitative PCR. This analysis revealed considerable differences in CEACAM6 expression depending on the cell line. While T84 cells showed the highest expression, the expression in HCT116 cells was four orders of magnitude smaller and close to the detection limit (Fig. 2A). SW480 and DLD-1 cells showed similar, intermediate expression (Fig. 2A). Interestingly, when these cell lines were analyzed for *CEACAM6* gene variants, only T84 and HCT116 cells, the two cell lines with the highest and lowest CEACAM6 expression, respectively, had unique genotype variants when compared with the other cell lines (Fig. 2B). While T84 cells were the only cells that had a unique genotype for rs10415946 and rs1805223 = p.Pro42Pro, HCT116 had a unique genotype in SNPs rs11548735 = p.Gly239Val, rs2701 and rs10416839. SW480 and DLD-1 cells had identical *CEACAM6* genotypes and their CEACAM6 expression was nearly identical (Fig. 2A and 2B). A detailed analysis regarding linkage disequilibrium between the investigated *CEACAM6* SNPs stratified for CD, UC and controls is given in Tables 11, 12 and 13.

**Table 11:** Analysis for linkage disequilibrium between *CEACAM6* SNPs in patients with Crohn's disease.

	rs10415946	rs4803507	rs4803508	rs2701	rs10416839
**rs10415946**	*	*	*	*	*
**rs4803507**	0.65/0.96	*	*	*	*
**rs4803508**	0.12/0.58	0.09/0.59	*	*	*
**rs2701**	0.12/0.53	0.10/0.57	0.80/0.95	*	*
**rs10416839**	<0.01/0.03	<0.01/0.13	0.25/0.85	0.31/0.90	*

Values are given as r^2^/D′-measurements.

**Table 12:** Analysis for linkage disequilibrium between *CEACAM6* SNPs in patients with ulcerative colitis.

	rs10415946	rs4803507	rs4803508	rs2701	rs10416839
**rs10415946**	*	*	*	*	*
**rs4803507**	0.70/0.98	*	*	*	*
**rs4803508**	0.15/0.65	0.11/0.67	*	*	*
**rs2701**	0.14/0.61	0.10/0.60	0.87/0.97	*	*
**rs10416839**	<0.01/0.09	<0.01/0.11	0.29/0.89	0.33/0.91	*

Values are given as r^2^/D′-measurements.

**Table 13:** Analysis for linkage disequilibrium between *CEACAM6* SNPs in controls.

	rs10415946	rs4803507	rs4803508	rs2701	rs10416839
**rs10415946**	*	*	*	*	*
**rs4803507**	0.68/0.98	*	*	*	*
**rs4803508**	0.15/0.63	0.09/0.60	*	*	*
**rs2701**	0.14/0.60	0.09/0.57	0.81/0.94	*	*
**rs10416839**	0.01/0.12	<0.01/0.06	0.23/0.84	0.29/0.90	*

Values are given as r^2^/D′-measurements.

## Discussion

In summary, our study represents the first detailed analysis of *CEACAM6* SNPs regarding disease susceptibility and phenotypic consequences in IBD patients. Compared to previous GWAS, our study had a more complete coverage of the *CEACAM6* gene region (see details in Table 14). Overall, we did not observe a significant influence of the investigated *CEACAM6* SNPs on CD and UC susceptibility. Moreover, a detailed haplotype analysis did not reveal significant associations with IBD susceptibility. CEACAM6 has recently shown to act as a receptor for AIEC suggesting an important role in bacterial colonization of the ileal mucosa in CD patients [19]. However, none of the investigated *CEACAM6* SNPs was associated with ileal or ileocolonic CD.

**Table 14:** Coverage of the *CEACAM6* gene region by the Illumina Hap300 chip and the Affymetrix 500 k chip utilized in previous genome-wide association studies (GWAS).

Chromosomal position (bp) of the *CEACAM6* SNP	Position in the *CEACAM6* * gene	*CEACAM6* SNPs analyzed in our study	*CEACAM6* SNPs covered by the Illumina Hap300 chip	*CEACAM6* SNPs covered by the Affymetrix 500k chip
46,948,446	upstream		rs3764577	
46,950,899	upstream	rs10415946		
46,952,409	intragenic	rs1805223 = P42P		
46,953,560	intragenic			rs3795018
46,954,095	intragenic			rs11669653
46,954,731	intragenic		rs3795020	
46,955,390	intragenic	rs4803507		
46,956,489	intragenic	rs4803508		
46,957,729	intragenic	rs11548735 = G239V		
46,957,793	intragenic	rs7246116 = H260H		
46,962,846	intragenic		rs10413359	
46,966,939	intragenic	rs2701		rs2701
46,970,128	downstream			rs6508997
46,972,172	downstream	rs10416839	rs10416839	

*Position of the *CEACAM6* gene on chromosome 19: 46,951,341 bp to 46,967,953 bp.

Interestingly, a recent study demonstrated that the defect in CEACAM family members in intestinal epithelial cells isolated from CD patients appears to be related to the aberrant nuclear localization of the transcription factor SOX9 [39] which regulates cell proliferation and is required for Paneth cell differentiation in the intestinal epithelium [40], [41]. However, ileal CD is characterized by a specific decrease in Paneth cell alpha-defensins and defective Paneth cell-mediated host defense [42] which has been linked to the *NOD2* genotype [43], although this finding is opposed by the results of a recent study [44], and additional modifiers of Paneth cell function such as XBP1 are involved [45]. Therefore, one might speculate whether the role of CEACAM6 in ileal bacterial colonization is regulated via SOX9 expression implicating defective Paneth cell function in patients with small bowel CD. Given the association of defensin secretion with the *NOD2* genotype [43] and the findings of numerous previous studies including studies from our IBD center demonstrating ileal disease localization in CD patients with *NOD2* mutations [37], [46], [47], we also tested for potential gene-gene interaction of *CEACAM6* and *NOD2*. However, we found no evidence for epistasis between these two genes regarding CD susceptibility. Further functional studies analyzing the complex interaction of intestinal CEACAM6 expression and bacterial adherence in the gut particularly of CD patients carrying *CEACAM6* variants will therefore be required. Given the important role of *ATG16L1* in Paneth cell development of the terminal ileum and the role of *IL23R* in the development of proinflammatory Th17 cells, we also analyzed for epistasis of these two genes with *CEACAM6* but were unable to find evidence for significant epistasis of these genes regarding CD susceptibility.

Interestingly, a recent study indicated that *CEACAM6* and a regulatory element near the 3′ end of *CEACAM3* are associated with disease severity in patients with cystic fibrosis [48]. However, a previous study in IBD patients suggested that heterozygous carriers of the ΔF508 mutation in the *CFTR* gene, the main susceptibility gene for patients with cystic fibrosis, might exert a protective effect in CD [49], suggesting opposing effects of genetic risk loci for cystic fibrosis and IBD.

In the meta-analysis of Barrett et al. [50], a SNP (rs4807569) within the chromosomal region 19q13, in which the *CEACAM6* gene is located, was weakly associated with CD, but this association could not be confirmed in a replication cohort. In the recent meta-analysis of Franke et al. [51], two SNPs (rs 736289 and rs281376) within this region were strongly associated with CD. However, the distance between these SNPs and the *CEACAM*6 gene is 9 and 7 megabases, respectively, and thus, the disease causing variant within this region remains to be identified.

*CEACAM6* is also a major target gene for Smad3-mediated TGF-β signaling [52]. Since Smad3 differentially regulates the induction of regulatory and inflammatory Th17 cell differentiation [53], which are key players in the IBD pathogenesis [54], further investigations analyzing Th17 cell differentiation in IBD patients carrying *CEACAM6* variants might also be of high interest. Moreover, very recent evidence from studies in mice demonstrated that colonization of the small intestine with a single commensal microbe, segmented filamentous bacterium (SFB), is sufficient to induce Th17 cells in the lamina propria [55]. These SFB adhere tightly to the surface of epithelial cells in the terminal ileum of mice with Th17 cells but are absent from mice that have few Th17 cells [55]. Further studies will have to characterize if SFB adherence is mediated (similar to AIEC adherence) by CEACAM family members.

In summary, we performed the first systemic analysis of *CEACAM6* gene variants in IBD patients. Despite the great importance of CEACAM6 as receptor for AIEC on the ileal mucosa of CD patients, we were unable to demonstrate a specific role of *CEACAM6* variants in IBD susceptibility. Furthermore, there was no evidence for an association with ileal CD or for epistasis with *NOD2*, *IL23R*, and *ATG16L1* variants in CD susceptibility. Further functional studies will be necessary to elucidate how *CEACAM6* gene variants may modulate bacterial colonization in IBD patients. Even if this study was unable to find a role for *CEACAM6* gene variants in IBD susceptibility, the CEACAM6 protein is likely to be an important mediator of the pathogenesis of CD [56].
